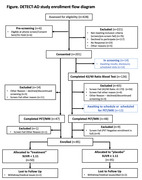# DETECT‐AD ‐ Digital Evaluations and Technologies Enabling Clinical Translation for Alzheimer’s Disease: Results of amyloid screening methodologies in a simulated anti‐amyloid clinical trial using digital biomarkers as primary outcome measures

**DOI:** 10.1002/alz.091154

**Published:** 2025-01-09

**Authors:** Jeffrey A Kaye, Nora Mattek, Amanda B Mar, Jennifer Marcoe, Elise J Hanna, Aimee Pierce, Yanan Shang, Kirsten M Wright, Jonathan A Wyatt, Daniel Schwartz, Sarah Gothard, Wan‐Tai M. Au‐Yeung, Joel S. Steele, Zachary T Beattie, Kevin Duff, Lisa C Silbert

**Affiliations:** ^1^ NIA‐Layton Aging & Alzheimer's Disease Research Center, Portland, OR USA; ^2^ Oregon Center for Aging & Technology (ORCATECH), Portland, OR USA; ^3^ Oregon Health & Science University, Portland, OR USA; ^4^ Portland Veterans Affairs Medical Center, Portland, OR USA; ^5^ NIA‐Layton Oregon Alzheimer's Disease Research Center, Portland, OR USA; ^6^ NIA‐Layton Oregon Alzheimer's Disease Research Center, Oregon Health & Science University, Portland, OR USA; ^7^ University of North Dakota, Grand Forks, ND USA

## Abstract

**Background:**

With the advent of anti‐amyloid therapies, identifying those with underlying amyloid burdens and detecting subsequent clinical effects of this AD pathology is critical. The DETECT‐AD study (ClinicalTrials.gov, NCT05385913) is a simulated secondary prevention anti‐amyloid clinical trial testing digital biomarkers as more sensitive and meaningful primary outcome measures. Clinically prodromal AD patients with estimable rates of AD progression based on Aβ PET status are enrolled as Aβ “positive” (higher amyloid burden) patients who predictably progress (as if receiving placebo) and Aβ “negative” patients progressing more slowly (representing the treatment group). Typical of current trials, participants are screened at entry to determine those with critical CNS amyloid burdens. Given the effort and cost of establishing AD phenotypes, efficient means for this screening are needed. Here we report to date the results of examining the screening process using blood‐based amyloid determinations for identifying florbetapir PET imaging‐based, amyloid status leading to enrollment of prodromal AD trial participants.

**Method:**

Participants were initially screened by telephone using inclusion/exclusion criteria questionnaires. If passing this phase, participants underwent in‐person cognitive and neurological examinations and clinical blood tests; if passing this phase, an MRI/amyloid PET scan was performed; if passing this last step, the digital technologies were deployed in‐homes. The first 47 participants used this protocol. The subsequent 140 participants underwent a blood amyloid test (plasma Aβ42:40) first, and then the remaining procedures if intermediate or high risk for CNS amyloid (Aβ42:40 < 0.170).

**Result:**

A Consort Diagram shows the study results (Figure). As of 1/3/2024, 85/100 have been enrolled with mean(SD): follow‐up=59.4(21.3) weeks; age=78.2(6.5); 65% women; MoCA=25.0(2.5); PET SUVR=1.14(0.23), range: 0.806‐2.18. Without blood amyloid pre‐screening, 26% of participants were PET amyloid positive (SUVR≧1.11). With the blood amyloid screening, 52% were PET amyloid positive.

**Conclusion:**

A simple telephone screen and blood amyloid test significantly improved the yield of amyloid PET positive participants in a simulated clinical trial, which can reduce costs, personnel effort, and participant burden and risk. Follow‐up will suggest how additional simple clinical, digital, and blood‐based biomarkers may further aid in identifying well‐defined target populations for clinical trials.